# 外周血游离DNA的动态变化预测TKI治疗*EGFR*突变肺腺癌患者的疗效

**DOI:** 10.3779/j.issn.1009-3419.2019.09.03

**Published:** 2019-09-20

**Authors:** 雨光 宋, 硕 王, 艳杰 赵, 妮 姜, 国良 乔, 静 赵, 岩 邸, 小莉 王, 军 任

**Affiliations:** 100038 北京，首都医科大学附属北京世纪坛医院肿瘤内科 Department of Medical Oncology, Beijing Shijitan Hospital, Capital Medical University, Beijing 100038, China

**Keywords:** 肺肿瘤, 表皮生长因子受体, 酪氨酸激酶抑制剂, 二代基因测序, 循环肿瘤DNA, Lung neoplasms, Epidermal growth factor receptor, Tyrosine kinase inhibitors, Next gene sequencing, Circulating tumor DNA

## Abstract

**背景与目的:**

有表皮生长因子受体（epidermal growth factor receptor, *EGFR*）突变的非小细胞肺腺癌，患者在给予酪氨酸激酶抑制剂（tyrosine kinase inhibitors, TKIs）的治疗中获得非常好的疗效，但绝大多数患者都会出现耐药，使得发现出现耐药的时间及可能耐药机制的检测有着越来越大的意义，目前二代基因测序方法（next generation sequencing, NGS）的出现使其成为可能。本文拟通过研究靶向治疗前后有*EGFR*突变的非小细胞肺癌循环肿瘤DNA（circulating tumor DNA, ctDNA）突变频率及突变谱的变化来监测靶向治疗效果。

**方法:**

本中心入组22例通过组织活检或外周血ctDNA检查出*EGFR*突变的患者，分别于治疗前、服用TKI后2个月及临床进展时收集患者的外周血8 mL行ctDNA测序。

**结果:**

*EGFR*敏感突变患者应用靶向药物治疗效果显著，*EGFR*敏感突变的患者在用TKI治疗后，相比治疗前突变基因突变丰度明显降低（*P*=0.015, 3）; 患者的中位无进展生存期较长（无进展生存时间=390 d）。同时我们发现伴随*TP53*基因突变时应用针对*EGFR*敏感突变的靶向药物治疗效果欠佳（中位无进展生存时间为120 d *vs* 630 d，*P*=0.000, 2）。

**结论:**

*EGFR*敏感突变的患者在用TKI治疗后，突变基因突变丰度明显降低的患者的生存期更长（*P* < 0.05），突变丰度减低不明显或伴有其他突变者预示着TKI耐药。

## Abstract (en)

**Background and objective:**

The tyrosine kinase inhibitors (TKI) treatment of non-small cell lung cancer (NSCLC) with epidermal growth factor receptor (*EGFR*) mutation may have a positive effect, but most patients may develop drug resistance, therefore, the detection of the developing time in drug resistance and the research of the mechanism of drug resistance are need to be solved. While the emerge of next generation sequencing (NGS) have make it possible. The aim of this study is to monitor the efficacy of targeted therapy by studying the variation of circulating tumor DNA (ctDNA) mutation frequency and gene mutation spectrum through the targeted therapy.

**Methods:**

Our center enrolled 22 patients with *EGFR* mutation detected by tissue or peripheral blood, and collect 8 mL of peripheral blood of the patients for ctDNA sequencing in different phases, before systematic prior treatment, followed-up by 2 months and disease progression after TKI administration.

**Results:**

Patients with E*GFR* gene mutation may acquire a longer median survival time after receiving targeted drug therapy, due to the drop of mutation abundances, while the therapy may have a minor effect in patients which their mutation abundances have slightly decreased compared to the statistics before the cession (*P*=0.015, 3). The significantly reduced group median progression was associated with a longer survival [progression free survival (PFS)=390 d]. At the same time, we found out that when related to *TP-53* gene mutation, the effect of targeted drug therapy for *EGFR*-sensitive mutation was unsatisfactory (the median PFS was 120 d compared with 630 d, *P*=0.000, 2).

**Conclusion:**

Patients who has lower mutation abundance with *EGFR* sensitive mutations after TKI treatment may have a longer survival period (*P* < 0.05), and the mutation abundance were not significantly dropping or accompanied by other mutations may indicating TKI resistance.

根据2018年全球肿瘤发病率以及死亡率统计，肺癌仍然是全球发病率以及死亡率最高的癌种，新诊断的数量占所有癌种数的11.6%，死亡人数占所有癌种中第一位^[1]^，而在亚洲地区，尤其是在中国，在所有肿瘤中肺癌的数量仍然高居第一名。其中非小细胞肺癌（non-small cell lung cancer, NSCLC）在肺癌中占比高达80%，肺癌患者中约55%的病例在就诊时已经发生远处转移，5年生存率仅为10%-15%。最近十几年里，表皮生长因子受体酪氨酸激酶抑制剂（epidermal growth factor receptor-tyrosine kinase inhibitors, EGFR-TKIs）等相应靶向药物已经成为晚期非小细胞肺腺癌重要的治疗方式之一，且已有多项临床研究^[2]^证实TKI的靶向治疗能显著降低伴有*EGFR*基因突变的晚期NSCLC患者疾病进展或死亡风险、改善患者生活质量; 对有*EGFR*敏感突变且对一代TKI治疗有效的患者中位无进展生存期为10个月-16个月^[3]^; 但是第一代TKI的耐药一直是临床难以解决的问题，目前已有相关研究^[4]^认为耐药的主要原因是*T790M*基因改变的结果^[5]^，而三代TKI奥希替尼的成功上市解决了部分*T790M*基因突变所致耐药的问题，而除此以外，还有一些基因，比如*ERBB2*、*ERBB3*等表达的蛋白扩增; *BRAF*、*MAP2K1*、*PI3KCA*以及*KRAS*基因的错义突变; *PTEN*基因的失活和*ALK*融合基因的表达都会对TKI治疗*EGFR*突变的患者产生影响，最终导致耐药从而病情进展^[6]^。而一些研究^[7]^中，研究者通过采取应用TKI治疗*EGFR*突变肺癌患者不同时间段的外周血，通过cfDNA的基因检测发现12例患者中6例患者T790M在治疗过程中逐渐升高，而其余的患者没有发现*T790M*基因的产生，2例患者中出现了上皮间质转换（epithelial-mesenchymal transition, EMT）现象，而其中的1例有RB1失活，1例*RB1*基因出现突变; 这说明每一例患者获得性耐药的机制都是不同的，而在肿瘤发生的不同时间，基因的变化也是不尽相同的。

为了更好地解决上述问题，我们需要在治疗过程中检测患者的基因水平的变化，明确有无新的耐药基因的产生，同时通过基因水平的变化检测TKI治疗的疗效，但是在治疗进展后很难应用传统的穿刺方式来获得患者的病理组织。循环肿瘤DNA（circulating tumor DNA, ctDNA）测序技术的发展给上述问题带来了很好的解决方案。对比前后的ctDNA突变变化可以作为一个潜在TKI治疗效果的指标^[8]^。

基于此，我们应用ctDNA测序技术比较应用TKI治疗前后的基因谱组成以及突变频率变化，从而预测TKI治疗的疗效。同时我们观察到一些新发的耐药基因产生，比如T790M、P53、JAK等从而影响TKI治疗的疗效。

## 1 资料与方法

### 1.1 试验设计

我们进行了一项前瞻性单中心研究，以测试ctDNA在评估*EGFR*突变NSCLC患者治疗反应和监测EGFR状态的有效性。入组合格标准包括具有EGFR突变的肺腺癌、年龄18岁-75岁，体力状况（performance status, PS）评分0分-2分，临床分期Ⅲb期或Ⅳ期患者。并签署书面知情同意书及同意用TKI治疗的患者; 排除标准：PS评分 > 2分，年龄 > 75岁及选择TKI治疗以外治疗的患者。2016年11月-2018年8月，共有22例患者参与了这项研究。每例患者根据自愿接受三种已在中国上市的TKI药物中的任何一种（易瑞沙，特罗凯; 凯美纳），在EGFR-TKI治疗期间，患者外周血液样本（每8 mL）分别在治疗前1周及治疗后2个月及临床评效进展后分别各采集一次进行ctDNA测序分析应用。肿瘤对治疗的反应是通过计算机断层扫描（computed tomography, CT）确定的，并根据实体瘤疗效评价标准（Response Evaluation Criteria in Solid Tumors, RECIST）进行评估，分为完全缓解（complete response, CR）、部分缓解（partial response, PR）、稳定（stable disease, SD）以及病情进展（progressive disease, PD）。

### 1.2 血浆样品处理与ctDNA提取

用EDTA真空采血管（BD Diagnostics, Franklin Lakes, NJ, USA）采集患者8 mL外周血并在4 h内完成血浆分离。血浆中ctDNA的提取使用的是QIAamp Circulating Nucleic Acid Kit（Qiagen, Hilden, Germany）。DNA浓度的检测使用QubitdsDNAHS（High Sensitivity）Assay Kit（Invitrogen, Carlsbad, CA, USA），并利用Agilent 2100 BioAnalyzer and the DNA HS kit（AgilentTechnologies, Santa Clara, CA, USA）检测ctDNA的片段分布。

### 1.3 ctDNA测序范围

体细胞变异基因检测范围包含全部外显子区域基因（288个基因的4, 728个外显子）; 其他相关基因728个基因的1, 074个编码区域。

### 1.4 统计学分析

本试验统计学分析都是基于Graphpad Prism软件获得，ctDNA突变频率变化采用同一患者治疗前后对比配对*t*检验所获得，*P* < 0.05代表差异有统计学意义。采用*Kaplan-Meier*方法进行生存分析。应用SPSS 21.0 统计软件进行统计学处理，应用Graphpad Prism软件绘图。计量资料以均数±标准差（Mean±SD）表示，两组间比较采用卡方检验，我们应用*Cox*比例回归模型进行无进展生存时间（progression free survival, PFS）的评估，采用单因素变量即一次采用一个因子，然后任何所有因素的多变量模型来分析潜在预后变量。

## 2 结果

### 2.1 患者临床特征

参加本试验研究的22例患者中半数患者（50%）应用吉非替尼治疗，其余依据患者意愿6例应用厄洛替尼，5例应用埃克替尼治疗。治疗前后收集了外周血进行循环肿瘤DNA测序，22例患者详细信息如表 1所示，本试验入组患者中位年龄为63岁（范围44岁-94岁），入组女性患者占绝大多数，其中18例患者的体力状况较好（PS=0分或1分）。治疗前的测序结果是EGFR 21位点突变14例，EGFR 19位点缺失突变5例。

**1:** 22例患者的临床特征比较 The comparison of clinical features in 22 cases

Group	Male	Female	Total
Quantity	6	16	22
EGFR19 deletion in exon	5	9	14
*EGFR21* mutation in exon	1	7	8
*TP53* mutation	2	7	9
CR1	1	5	6
PR1	0	5	5
SD1	2	4	6
PD1	3	2	5
CR2	1	1	2
PR2	0	0	0
SD2	0	0	0
PD2	5	15	20
JAK2	1	1	2
PS0	3	12	15
PS1	2	3	5
PS2	1	1	2
Ⅲb	5	15	20
Ⅳ	1	1	2
CR1, PR1, SD1, PD1 was evaluated after being taken TKI in 2 months; CR2, PR2, SD2, PD2 was evaluated after being taken TKI in 24 months or clinical progression. CR: complete response; PR: partial response; SD: stable disease; PD: progressive disease. EGFR: epidermal growth factor receptor.			

### 2.2 基因突变情况

治疗中1例患者出现T790M突变，1例患者出现L861Q突变，7例患者出现*TP53*基因伴随突变，2例患者出现JAK2伴随突变。随访2个月时5例PD，6例SD，PR及CR共11例; 24个月随访结果2例CR，20例PD，其中死亡9例。统计学结果如图 1所示22例患者基于外周血检测的*EGFR*突变所应用靶向药物治疗后中位PFS为390 d（范围：40 d-1, 500 d），而这也与组织学检测结果后应用靶向药物治疗时间相似（吉非替尼13.7个月，厄洛替尼14个月）（图 1）。

**1:**
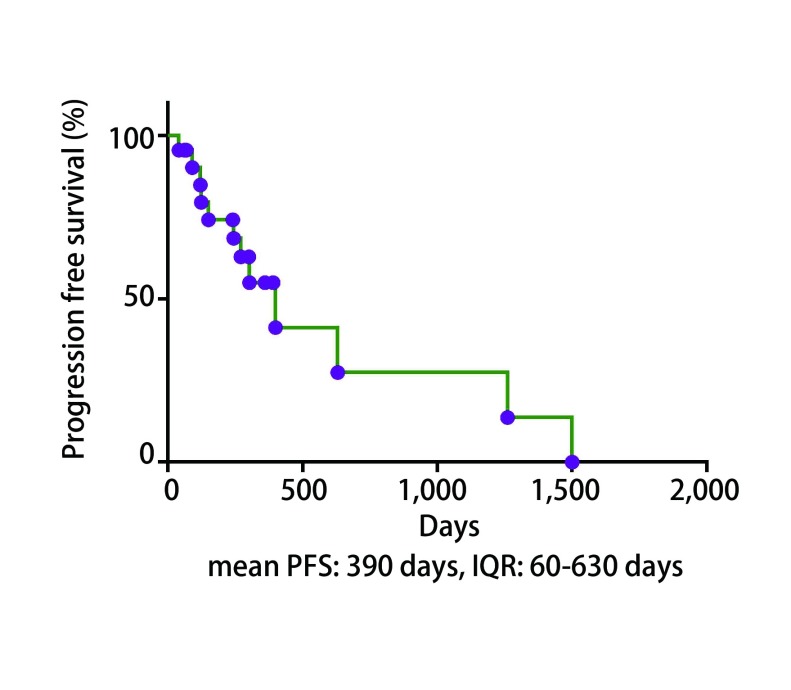
*EGFR*敏感突变组的中位平均生存期。 Median survival time of *EGFR* sensitive mutation group

### 2.3 对比前后外周血中ctDNA中*EGFR*突变基因丰度变化及出现其他伴随突变可以预测靶向药物治疗疗效

为了解循环血中ctDNA是否可以预测靶向药物治疗疗效，我们将22例患者服用靶向药物后2个月或临床进展后再次抽取了外周血行ctDNA测序，从图 2中我们看出相比治疗前，15例患者应用靶向药物治疗后出现了*EGFR*突变频率丰度明显下降（*P*=0.015, 3），其PFS明显延长，达630 d，*P*=0.042，通过基因检测发现具有TP53及JAK2伴随突变的患者预后较差。如图 3所示，如果患者伴随*TP53*突变的情况下相比未伴随突变生存期明显降低（中位PFS为160 d *vs* 630 d，*P*=0.000, 2）。*JAK2*突变因数量较少，未给予统计学处理。如图 4所示，其中7例患者再检测时*EGFR*基因丰度变化不大，*P*=0.67，临床评效多为稳定。在有*TP53*突变的患者，随着TKI的治疗，其丰度逐渐升高，且临床疗效较差，提示本部分患者可能先天对TKI耐药（图 2-图 4）。

**2:**
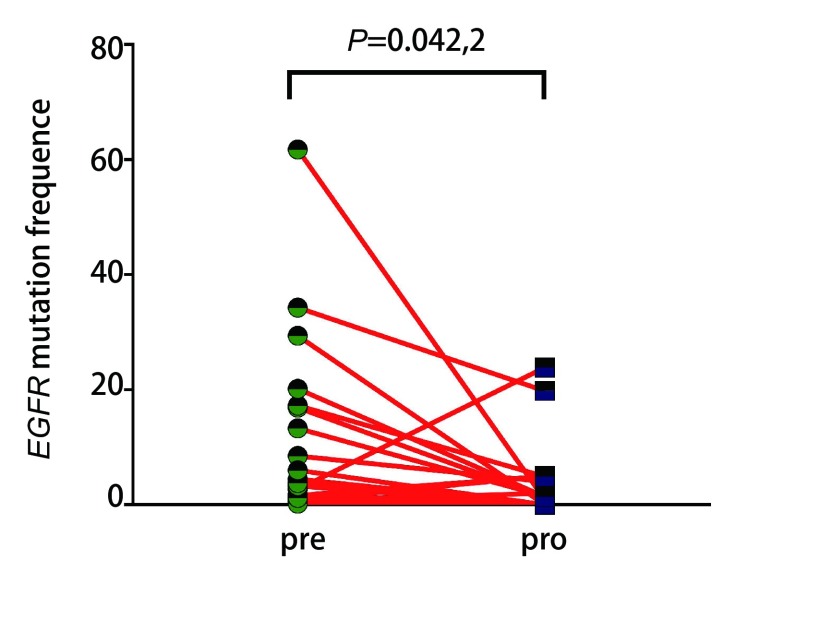
治疗前后*EGFR*突变基因丰度变化图 The comparative abundance of *EGFR* sensitive mutation before and after treatment

**3:**
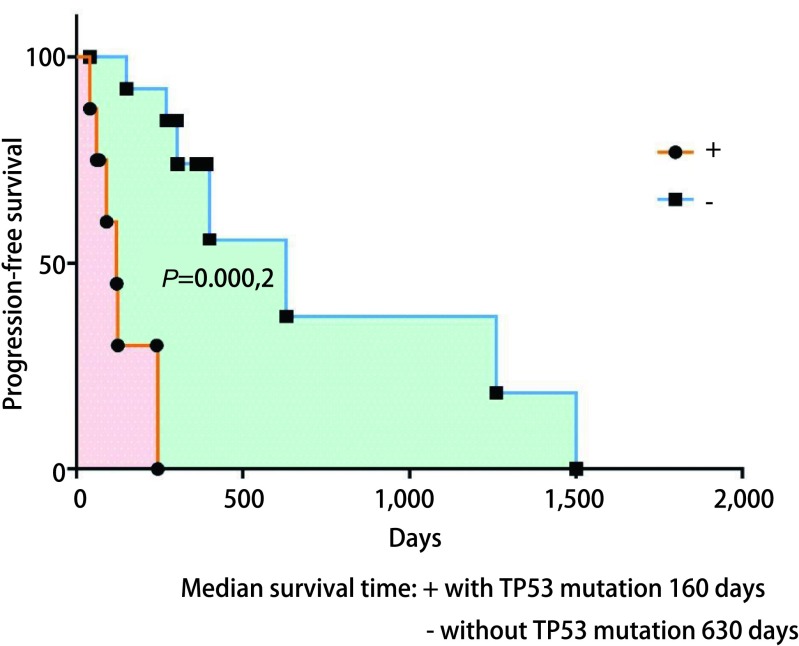
伴有或不伴有TP-53伴随突变患者的PFS Progression-free survival of cases with or without *TP53* mutatio

**4:**
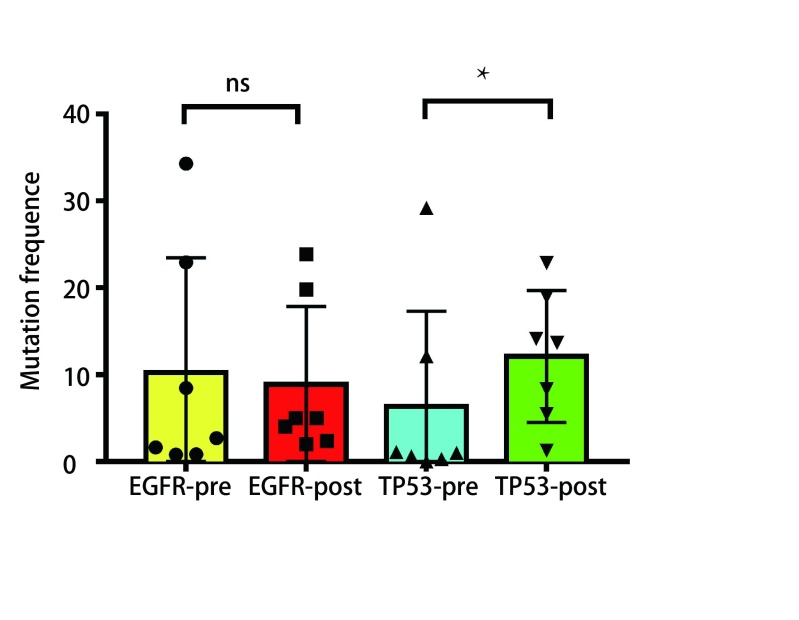
*EGFR*及*TP-53*突变基因治疗前后丰度变化图 The comparative abundances of *EGFR* and *TP-53* mutation before and after treatment

## 3 讨论

目前已有较多的研究证明了在NSCLC治疗中，可以基于ctDNA的癌症基因突变的结果给予靶向治疗。有研究^[8]^中65%的患者存在ctDNA突变，并给予有针对性的治疗方案，包括26%的患者可以行动态的癌症基因检测。有研究^[9]^于2017年在《*Nature*》上发表了一篇回顾性文章，其分析了100例NSCLC术后的患者，采用游离DNA方法预测治疗效果，结果ctDNA对复发的预测敏感性是93%，能够比传统影像及肿瘤标志物等检测方法早11个月发现进展。

在癌症患者血浆中检测到携带肿瘤特异性序列改变的DNA片段，即ctDNA目前被认为是组织活检的合理补充^[10]^。最近，ctDNA在监测肿瘤负担、最小残留疾病和获得性耐药中的作用正在深入研究中，在Abbosh等^[11]^的报告中，ctDNA的变化和EGFR-TKI治疗的临床反应之间有良好的相关性。然而，具有EGFR激活突变的肿瘤是由含有多种*EGFR*基因等位基因组合的异质性肿瘤细胞克隆组成的，包括激活突变体、野生型和原发T790M突变型^[12]^，它们的比值在EGFR-TKI治疗过程中随时间变化。因此，动态监测ctDNA变化可以观察到对EGFR-TKI的反应，并及时反映出每个克隆的变化。此外，EGFR-TKI的治疗对*EGFR*基因的影响无疑会诱导肿瘤细胞的克隆进化，如果是这样的话，ctDNA将为我们提供一个工具来观察肿瘤的克隆结构和它们随时间进化的动态，并可以作为评估对治疗或耐药性发展的反应。在我们的研究中，可以看出EGFR-TKI治疗过程中有多基因片段ctDNA的动态变化，而这些基因的动态变化，对药物敏感突变频率丰度下降预示着治疗有效，通过上述试验结果我们初步认为ctDNA特定靶点的基因突变频率丰度变化可以预示靶向治疗的疗效。

在我们的研究发现在2例患者治疗中出现*JAK2*（p.V617F）基因突变，约占测试的1%病例。在肺癌的治疗中，JAK-STAT途径的作用得到越来越多的人认识^[13]^。有研究^[14]^描述JAK-STAT途径的活动：有*EGFR*突变的患者在给与TKI治疗中，尤其在出现EGFR-TKI抵抗时，常出现*JAK2*伴随突变，此时给与JAK2抑制剂后，能使耐药细胞再次对TKI治疗有效。而*TP53*基因的伴随突变在许多研究中亦有报道，可以影响EGFR靶向治疗的疗效^[15, 16]^。有研究^[17]^在105例患者中发现了43例*TP54/EGFR*双突变患者（占所有检测的41%）。其PFS和OS的缩短都与TP53状态有关。在Shepherd^[18]^的研究中纳入60个晚期肺腺癌患者，其中24个是*TP53*突变型; 36例TP53野生型，在获得第一代EGFR TKI治疗后，观察到在EGFR TKI和TP53突变（HR=1.74, 95%CI: 0.98-3.10, *P*=0.06）之间有一个不显著的影响趋势，*TP53*突变（*n*=17）时，PFS显著缩短（HR=1.91, 95%CI: 1.01-3.60, *P*=0.04）; 但在后续在使用T790M抑制剂治疗的11例可评估患者中，没有发现显著差异（TP53/3: 100%, WT 7/8: 88%）。其结论认为双*TP53/EGFR*突变的患者，在接受EGFR TKI治疗时，反应率降低，PFS的缩短。我们的研究虽然样本量较小，但也看出同样的趋势。

通过以上研究我们认为通过NGS液体活检方法，检测EGFR的ctDNA变化，可以较准确的反映出*EGFG*敏感突变的NSCLC对酪氨酸激酶抑制剂的治疗效果，其敏感基因突变丰度的快速降低可能预示着预后良好。而通过这种测序手段我们也能监测治疗过程中肿瘤的空间异质性变化，那些临床评效为PR/CR的病例，其敏感突变的丰度快速减低，可能预示着肿瘤细胞以单一*EGFR*突变克隆为主; 临床评效为SD/PD的病例，其敏感丰度变化不大或反而升高及伴随较多其他突变，预示着癌症细胞的克隆较多。我们后续将进一步扩大样本量来进一步验证上述结论，并随着其他相关基因通路的研究结果，根据ctDNA的检测结果制定更加精准的肿瘤个体化治疗方案，并为EGFR-TKI治疗耐药后的其他治疗提供依据。
